# Gastric schwannoma

**DOI:** 10.1002/ccr3.2747

**Published:** 2020-02-21

**Authors:** Sreenath Meegada, Nooman Gilani, Andrei Balandin, Rajanshu Verma

**Affiliations:** ^1^ UT Health East Texas/Christus Good Shepherd Medical Center Longview TX USA; ^2^ Banner Thunderbird Medical Center Glendale AZ USA; ^3^ Ironwood Cancer and Research Center Glendale AZ USA; ^4^ UTHSC College of Medicine Memphis TN USA

**Keywords:** gastric schwannoma, nausea, stomach mass, vomiting

## Abstract

Gastric Schwannomas are rare benign slow‐growing tumors and warrant treatment/resection only when symptomatic. Watchful waiting is recommended for incidental or asymptomatic schwannomas.

## Graphical abstract

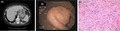

An 84‐year‐old lady with a 4‐year history of severe acid reflux, peptic ulcer disease presented with 1‐month history of epigastric abdominal pain, nausea, and vomiting. There was no history of weight loss, foreign travel, or sick contacts. Computed tomography of the abdomen done in emergency room showed a 4 × 4.1 × 4.1 cm mass in the gastric fundus of indeterminate nature (Figure 1A; scale). Esophagogastroduodenoscopy confirmed the findings of a 4‐5 cm hard submucosal gastric fundus mass without any stigmata of recent bleeding (Figure 1B). As initial biopsies were inconclusive, patient underwent partial gastrectomy for removal of gastric mass.^1^ Histopathology showed fascicular growth of neoplastic Schwann cells with palisading nuclei consistent with a diagnosis of gastric schwannoma (Figure 1C). Immunohistochemical stains revealed S100(+), CD117(−), DOG1(−), CD34(−), SMA(−), and Desmin(−).^2^ This immunophenotypic pattern supported neural differentiation. S100 stain is a marker of neural tissue and is positive in all neoplastic Schwann cells.

**Figure 1:**
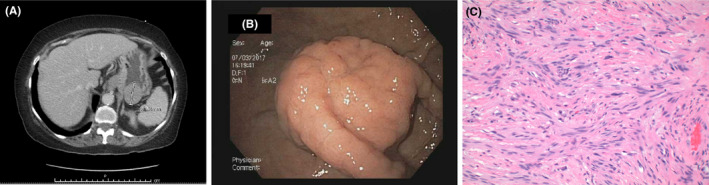
A, CT scan abdomen showing Gastric Schwannoma. B, Gastric Shwannoma on endoscopy. C, Hematoxylin and Eosin stain of Gastric Schwannoma

Schwannoma (also known as neurilemmoma) is a benign tumor made up of Schwann cells primarily affecting the peripheral nerves. Given their nonmalignant potential, no further treatment other than resection of a symptomatic Schwannoma is required. Stomach is an unusual location for presentation of a Schwannoma.

## CONFLICT OF INTEREST

None declared.

## AUTHOR CONTRIBUTIONS

SM: helped in writing the manuscript, revised submission, did literature search, and is corresponding author. NG: did endoscopy, and actively managed the case and guided in writing manuscript. AB: is Hematology‐Oncologist who was actively involved in patient care, helped in getting pathology slides, and gave valuable suggestions in writing the clinical image. RV: took care of the patient through the hospital course, helped in writing manuscript, took pictures of pathology, endoscopy slides, and did final proof reading of the submission.
